# Characterisation of Genome-Wide Association Epistasis Signals for Serum Uric Acid in Human Population Isolates

**DOI:** 10.1371/journal.pone.0023836

**Published:** 2011-08-19

**Authors:** Wenhua Wei, Gibran Hemani, Andrew A. Hicks, Veronique Vitart, Claudia Cabrera-Cardenas, Pau Navarro, Jennifer Huffman, Caroline Hayward, Sara A. Knott, Igor Rudan, Peter P. Pramstaller, Sarah H. Wild, James F. Wilson, Harry Campbell, Malcolm G. Dunlop, Nicholas Hastie, Alan F. Wright, Chris S. Haley

**Affiliations:** 1 MRC Human Genetics Unit, Institute of Genetics and Molecular Medicine, Western General Hospital, Edinburgh, United Kingdom; 2 The Roslin Institute and R(D)SVS, University of Edinburgh, Roslin, Midlothian, United Kingdom; 3 Institute of Genetic Medicine, European Academy Bozen/Bolzano (EURAC), Bolzano, Italy - Affiliated Institute of the University of Lübeck, Lübeck, Germany; 4 Institute of Evolutionary Biology, University of Edinburgh, Edinburgh, United Kingdom; 5 Centre for Population Health Sciences, University of Edinburgh, Edinburgh, United Kingdom; 6 Croatian Centre for Global Health, University of Split, Split, Croatia; 7 Department of Neurology, General Central Hospital, Bolzano, Italy; 8 Department of Neurology, University of Lübeck, Lübeck, Germany; Institute of Cytology & Genetics SD RAS, Russian Federation

## Abstract

Genome-wide association (GWA) studies have identified a number of loci underlying variation in human serum uric acid (SUA) levels with the *SLC2A9* gene having the largest effect identified so far. Gene-gene interactions (epistasis) are largely unexplored in these GWA studies. We performed a full pair-wise genome scan in the Italian MICROS population (n = 1201) to characterise epistasis signals in SUA levels. In the resultant epistasis profile, no SNP pairs reached the Bonferroni adjusted threshold for the pair-wise genome-wide significance. However, *SLC2A9* was found interacting with multiple loci across the genome, with *NFIA* - *SLC2A9* and *SLC2A9* - *ESRRAP2* being significant based on a threshold derived for interactions between GWA significant SNPs and the genome and jointly explaining 8.0% of the phenotypic variance in SUA levels (3.4% by interaction components). Epistasis signal replication in a CROATIAN population (n = 1772) was limited at the SNP level but improved dramatically at the gene ontology level. In addition, gene ontology terms enriched by the epistasis signals in each population support links between SUA levels and neurological disorders. We conclude that GWA epistasis analysis is useful despite relatively low power in small isolated populations.

## Introduction

Serum uric acid is the final oxidation product of purine metabolism in humans. High serum uric acid (SUA) levels can lead to gout and is associated with cardiovascular diseases and diabetes [1], whereas low SUA levels may be associated with multiple sclerosis [2], [3]. High SUA levels are increasingly prevalent (reaching 15–20%) in many human populations and caused mainly by impaired renal excretion of urate [4]. Elevated urate is associated with insulin resistance [5] and neurological disorders such as Parkinson's disease [6], [7]. About 70% of SUA is excreted via the kidneys and the remainder is eliminated into the biliary tract and intestine, part of which is subsequently converted by colonic bacterial uricase to allantoin [4].

SUA level is a complex trait that is affected by environmental (e.g. diet and excessive body weight) and genetic factors with heritability estimates of 60–87% [8], [9]. Genome-wide association (GWA) studies so far have identified nine loci underlying SUA levels. Seven of these loci are membrane transporters suggesting that the genetic variation in urate transport proteins plays an important role [10], [11], [12]. The variants identified within the *SLC2A9* gene are the most significant genetic risk factors associating with low fractional excretion of SUA and explain 5.3% of the SUA level variance in women and 1.7% of the SUA level variance in men in the VIS population [12]. However, because each of the eight remaining loci carries moderate marginal effects, the nine identified loci together only explain 5.22% of the SUA level variation [11], suggesting there may be more genetic loci to be detected.

One possible source of the unexplained variation in SUA levels is gene-gene interaction (epistasis) [13], [14]. Epistasis remains largely unexplored in previous GWA studies of SUA levels due to computational and statistical challenges, e.g. the lack of widely accepted algorithms that are fast enough to effectively handle high density SNPs and map different forms of epistasis while keeping false positive rates under control [15]. With the advances in computing technologies (e.g. GRID computing), full pair-wise genome scans are beginning to be applied in GWA data analyses [16], [17]. Nonetheless, fundamental questions about the potential values of GWA epistasis studies of normal GWA populations remain to be answered. Therefore, we performed a full pair-wise genome association scan in the Italian MICROS study cohort [18] to generate a profile of epistasis signals in SUA levels. The pair-wise genome scan used a regression-based comprehensive search algorithm that can detect epistasis signals with and without main effects [15], [19]. Epistasis signals detected in MICROS were tested for replication in a CROATIAN population combining the VIS [20] and KORCULA [21] study cohorts as well as the SOCCS (Phase 1) cohort [22]. Furthermore, we examined the gene ontology (GO) terms [23] enriched by the epistasis signals in both discovery and replication populations for any new insights into the genetic regulation of SUA levels.

## Materials and Methods

### Study cohorts and Ethics statement

The Italian MICROS cohort was recruited from the villages in South Tyrol [18]. This study was approved by the ethical committee of the Autonomous Province of Bolzano. The VIS [20] and KORCULA [21] cohorts were recruited from the islands of Vis and Korcula in Croatia respectively. This study was approved by the Ethical Committee of the Medical School, University of Zagreb and the Multi-Centre Research Ethics Committee for Scotland. The SOCCS (Phase 1) cohort was recruited in Scotland to study colorectal cancer [22]. This study was approved by the MultiCentre Research Ethics committee for Scotland. All participants gave written informed consent and were measured for a number of traits including SUA level, weight and height from which body mass index (BMI) values were calculated.

DNA samples were genotyped with Illumina Infinium HumanHap300v1/v2 or HumanCNV370v1 SNP bead microarrays and analyzed using the BeadStudio software. Quality control of the genotype data was performed for each cohort using the R/GenABEL package (Version 1.4.3) [24] based on a common set of criteria: individual call rate at 95%, SNP call rate at 98%, P value for deviation from Hardy-Weinberg equilibrium at 1.0e-10, minor allele frequency at 2%. The sample size and number of SNPs after the quality control were listed in Table 1 for each cohort.

**Table 1:** Summary information of each study cohort.*

	MICROS	VIS	KORCULA	CROATIAN	SOCCS
N	1201	895	877	1772	1097
#SNP	293913	300265	307712	283971	305449
SUA_median	5.17	5.09	4.84	NA	4.41
SUA_mean ± SD	5.32±1.42	5.24±1.59	4.91±1.29	NA	4.60±1.25
SUA and BMI correlation	0.38	0.35	0.38	NA	0.24
SUA polygenic heritability	0.325	0.288	0.228	0.287	NA

*: SUA level in mg/dL; CROATIAN combined VIS and KORCULA; NA: not available; the phenotypic correlation between SUA level and BMI was significant (P<2.2E-16) across MICROS, VIS and KORCULA.

### Statistical analysis

In each individual cohort the raw SUA levels were corrected for age, sex and BMI and normalised using the *rntransform* function that is implemented in the GenABEL package performing quantile normalisation of residuals from a generalized linear model analysis. The normalised SUA levels were then analysed using a linear mixed model to correct for polygenic effects and relatedness using the *polygenic* function in the GenABEL package and the resultant environmental residuals (i.e. pgresidualY) were used as the trait to test for association [25]. Polygenic heritability was estimated at the mixed model step. The CROATIAN combined population was created by merging the VIS and KORCULA study cohorts and the normalised SUA levels were corrected for study cohort, polygenic effects and relatedness as above and the resultant residuals were used as the trait for association tests.

A single SNP based GWA scan was performed in each population using a score test method (based on the additive model) implemented in the *mmscore* function in the GenABEL package. The consensus GWA threshold of 7.3 (−log_10_(5.0E-08)) was applied to identify GWA significant SNPs [26]. A full pair-wise genome scan was followed using regression models. Considering a pair of SNPs denoted as *SNP_1_* and *SNP_2_*, the following genetic models are used to detect epistasis where genotypes of each SNP (i.e. homozygote of the minor allele, homozygote of the major allele and heterozygote) were fitted as fixed factors [19], [27]:
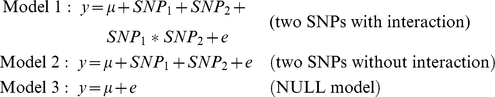
where *y* is the trait of interest, *μ* is the model constant, *SNP_1_* (or *SNP_2_*) is a fixed factor with three levels, SNP_1_*SNP_2_ is the interaction term, *e* is the random error term. The F ratio test of Model 1 against Model 3 is for the whole pair effect including interaction (i.e. *F_pair_*, 8 degrees of freedom). The F ratio test of Model 1 against Model 2 is for the interaction between the two SNPs (i.e. *F_int_*, 4 degrees of freedom). SNP pairs with missing joint genotype classes (i.e. considering three genotypes per SNP, at least one of the nine joint genotype classes of a SNP pair had no individuals) were not evaluated to reduced the risk of inflation of the type I error rate. P values were calculated based on the F distribution with relevant degrees of freedom and transformed in the −log_10_ scale (i.e. −log_10_P_pair_ for the *F_pair_* test, −log_10_P_int_ for the *F_int_* test). We applied the same −log_10_ scaled thresholds for both the *F_pair_* and *F_int_* tests to control the type I error rate [15].

Genome-wide significance thresholds (all in the −log_10_ scale) were derived based on Bonferroni correction for multiple tests, i.e. the 5% nominal P value corrected by the number of pair-wise tests. Considering 300,000 SNPs, a SNP-genome scan and a full pair-wise genome scan perform 3.0E+05 and 4.5E+10 association tests respectively, thus the genome-wide threshold is 11.95 (−log_10_(0.05/4.5E+10)) for the pair-wise genome scan. SNP-genome scans have been used to test epistasis for genome-wide significant signals specifically to increase the power of detection [15], [19], [28], [29] and thus are applied here to examine the interactions between each GWA significant SNP and all other SNPs genotyped. The actual GWA threshold for SNP-genome scans is calculated as −log_10_(0.05/3.0E+05/N) if there are N GWA significant SNPs.

A full pair-wise genome scan was performed in the MICROS population and SNP pairs with a certain interaction signal (i.e. −log_10_P_pair_>4.7 and −log_10_P_int_>3.2) were retained. The retained results were evaluated using the predefined thresholds to identify genome-wide significant epistatic signals. Each SNP in the retained results was annotated to the nearest gene within a window of 20 kilobases flanking the SNP based on the physical distances to either the start or end of transcription of genes (the distance is set to zero if the SNP is within a gene) without considering linkage disequilibrium (LD). A full pair-wise genome scan was also performed in the CROATIAN population as above to prepare input for GO enrichment analyses (see below).

### Replication, variance explained, and GO enrichment analysis

Epistatic pairs detected in the MICROS population were tested for replication in the CROATIAN population following the same procedures as above. The nominal threshold of 0.05 (or 0.05/K, if K epistatic pairs were tested) was used to claim significant replication for an epistatic pair (i.e. both replicated SNPs were exactly the same as the epistatic SNPs) because only one test was performed. When an epistatic pair was not replicated (e.g. due to missing genotype classes), we also tested the interactions between each of the adjacent SNPs of the first epistatic SNP and that of the second. In that case, the nominal threshold corrected by the actual number of tests was used to claim significant replication.

The *polygenic* function was also used to calculate the proportion of the phenotypic variance in SUA levels explained by epistatic pairs in the MICROS population. Because the quantile normalised SUA levels were based on the quantiles of the raw SUA levels, we used standardized SUA levels for variance calculation (i.e. the raw SUA levels were corrected for age, sex and BMI and then standardized (mean of 0 and variance of 1)). The standardized SUA levels were fitted into the full mixed model including polygenic effects and the identified SNP pairs. The difference of residual variance from a value of 1 is the proportion of phenotypic variance explained by the SNP pairs included.

A GO enrichment analysis was conducted for each of the MICROS and CROATIAN populations using the running mode of “Two unranked lists of genes” in GOrilla [23] where the full list of human genes was used as the background. We chose SNP pairs with a moderately high interaction signal (i.e. −log_10_P_pair_>6.5 and −log_10_P_int_>6.5) in each population and used the epistatic genes annotated from them as the target for the GO enrichment analysis to mine biological meanings from epistatic signals that were less significant. The GO terms enriched (P<1.0E-03) by the epistatic genes in each population were compared to identify replicated GO terms and then epistatic genes shared by each pair of replicated GO terms. The shared epistatic genes in the replicated GO terms were investigated further for a) associated biological functions or diseases and b) epistatic SNP pairs involved and their replication.

## Results

The mean SUA level and phenotypic correlation between SUA level and BMI were similar across the MICROS, VIS and KORCULA cohorts (Table 1). The polygenic heritability estimates varied from 0.228 (KORCULA) to 0.325 (MICROS), suggesting a different genetic background in each individual cohort. Using the normalised SUA residuals as the trait, a conventional GWA scan identified seven genome-wide significant SNPs in MICROS: rs737267, rs13129697, rs13131257, rs6449213, rs1014290, rs10805346 and rs733175 that were all annotated to the *SLC2A9* gene. The GWA scan for the CROATIAN population also identified seven genome-wide significant SNPs which were exactly the same as those in MICROS. Thus the genome-wide threshold of 7.62 (−log_10_(0.05/3.0E+05/7) was used for SNP-genome scans. The inflation factor λ (computed by regression in a quantile-quantile (QQ) plot, Figure S1) was 1.007 in both GWA scans, suggesting the family relatedness was well accounted for. In the SOCCS cohort 1097 unrelated individuals were measured for SUA and BMI where the mean SUA level (4.60±1.25) was lower than those in the three cohorts above (Table 1).

**Figure 1:**
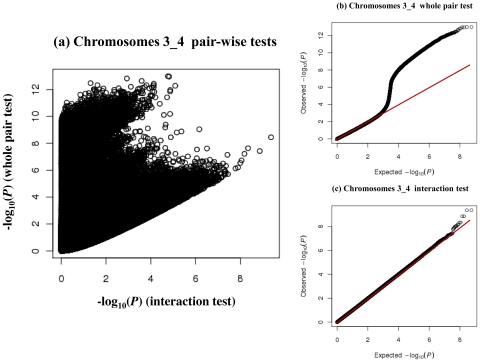
Plots of the P values of the pair-wise tests performed on chromosomes 3 and 4. SNP combinations with missing joint genotype classes were excluded. Left: scatter plot of the −log_10_P_pair_ against −log_10_P_int_ values of each SNP pair. Upper-right: QQ plot of the whole pair tests. Lower-right: QQ plot of the interaction tests.

The full pair-wise genome scan in the MICROS population tested 43 billion pair-wise SNP combinations where 11 billion pairs (26.3%) had missing joint genotype classes and hence were ignored in this study. We plotted the P values of all the *F_pair_* and *F_int_* tests performed on chromosomes 3 and 4 (the total number of pair-wise combinations is too large to plot at the genome level) to illustrate why the two tests are needed (Figure 1). Using only the *F_pair_* test (e.g. −log_10_P_pair_>11.95 may pick up SNP pairs with very weak interactions (e.g. −log_10_P_int_<3), whereas using only the *F_int_* test (e.g. −log_10_P_int_>7.62) could pick up those with weak whole pair effects when both SNPs had small marginal effects (e.g. −log_10_P_pair_<6) (Figure 1a). The QQ plot for the *F_pair_* tests (Figure 1b) showed an earlier departure (near the value of 3) from the expected line and that for the *F_int_* tests (Figure 1c) showed a late departure from the expected line. The QQ plots suggested that many pairs of SNPs on chromosomes 3 and 4 had strong whole pair effects attributing to the marginal effects in the *SLC2A9* region but only a few of them with interactions greater than expected under the null hypothesis. To further check the distributions of the test statistics, we randomly sampled 5000 SNPs from the MICROS genome and tested and stored all their pair-wise interactions with and without permutation. The two- sample Kolmogorov-Smirnov tests [30] found no significant difference (D = 0.0044, P>0.05) between the real (without permutation) −log_10_P_int_ distribution and the −log_10_P_int_ distribution under the null hypothesis.

**Figure 2:**
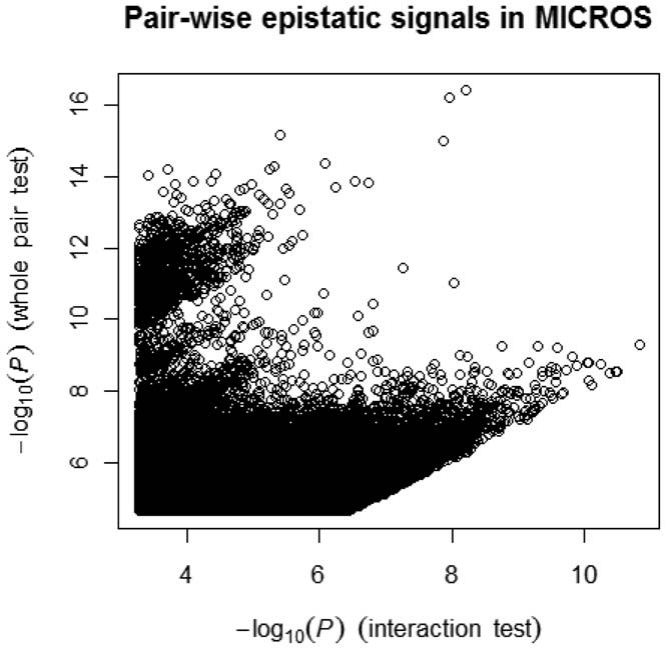
Profile of all retained epistatic pairs in the MICROS population. Each epistatic pair in the figure with −log_10_P_pair_>4.7 and −log_10_P_int_>3.2.

We plotted the pair-wise tests for all the SNP pairs (−log_10_P_pair_>4.7 and −log_10_P_int_>3.2, 212933 in total) retained from the full pair-wise genome scan (Figure 2). No SNP pairs reached the pair-wise genome scan threshold (i.e. −log_10_P_int_>11.95 along the x axis). However, we found a big cluster of SNP pairs with strong whole pair effects (i.e. −log_10_P_pair_>11.95 along the y axis) but weak to high interactions (i.e. 3.2<−log_10_P_int_<8.5 along the x axis). This cluster of SNP pairs all involved the *SLC2A9* gene as one would expect. Two pairs: rs12130085 (*NFIA*) – rs737267 (*SLC2A9*) and rs737267 (*SLC2A9*) – rs9316212 (*ESRRAP2*) were significant based on the threshold of 7.62 for SNP-genome scans (Table 2). These two pairs showed different interaction patterns (Figure S2) and jointly explained 8.0% (4.4% by rs727367 alone and 3.4% by interactions) of the variance of the standardised SUA levels in MICROS.

**Table 2:** *SLC2A9* involved epistatic pairs and replication.^1^

SNP_1_	chr_1_	gene_1_	SNP_2_	chr_2_	gene_2_	P_pair_	P_int_	MGC	CROATIAN replication^3^	SOCCS replication^3^
rs12130085	1	*NFIA*	rs737267	4	*SLC2A9*	4.1e-17 (16.38)	6.6e-09 (8.20)^2^	2	0.045	0.178
rs737267	4	*SLC2A9*	rs9316212	13	*ESRRAP2*	6.2e-17 (16.21)	1.1e-08 (7.97)^2^	2	0.048	0.238
rs733175	4	*SLC2A9*	rs1818116	5	*(-)*	3.8e-12 (11.43)	5.5e-08 (7.27)	7	0.130	0.227
rs733175	4	*SLC2A9*	rs137180	22	*SEZ6L*	8.0e-11 (10.09)	2.6e-07 (6.58)	4	0.035	0.155

1 : SNP_1_ (SNP_2_) – the first (second) SNP name; chr_1_ (chr_2_) – the chromosome where SNP_1_ (SNP_2_) locates; gene_1_ (gene_2_) – symbol of the gene annotated by SNP_1_ (SNP_2_); P_pair_ – P value of the whole pair test (−log_10_ P value in brackets); P_int_ – P value of the interaction test (−log_10_ P value in brackets); MGC – count of number of individuals in the minor joint genotype class; *(-)*: no gene annotation.

2 : genome-wide significant.

3 : Best replication including adjacent SNPs: the *SLC2A9* SNP is fixed and the other SNP is to replicate as itself or the first or second neighbour.

Considering only those with a moderately high interaction signal (−log_10_P_pair_>6.5 and −log_10_P_int_>6.5), in total 1326 SNP pairs involved 2063 unique SNPs of which 1148 (55.6%) were annotated to 910 unique genes, i.e. 1.5 pairs per gene. In contrast, 17 out of the 1326 SNP pairs involved the *SLC2A9* gene including 4 GWA significant SNPs rs733175, rs737267, rs13131257 and rs13129697 (Tables 2 and S1). Epistatic pairs listed in Table 2 were tested for statistical replication in the CROATIAN population. The two genome-wide significant epistatic pairs missed the SNP level replication: the −log_10_P_int_ values of the best replicated pairs of rs737267 – rs1779851 (adjacent to rs12130085) and rs737267 – rs17064136 (adjacent to rs9316212) were 1.35 and 1.32 respectively. Combining the MICROS and CROATIAN data did not make the two significant pairs stronger (−log_10_P_int_ was 2.32 and 2.19 respectively) which is in line with the replication results. The two less significant *SLC2A9* epistatic pairs also missed the SNP level replication in the CROATIAN population (Table 2). The four epistatic pairs in Table 2 also failed to achieve exact replication in the SOCCS cohort.

**Figure 3:**
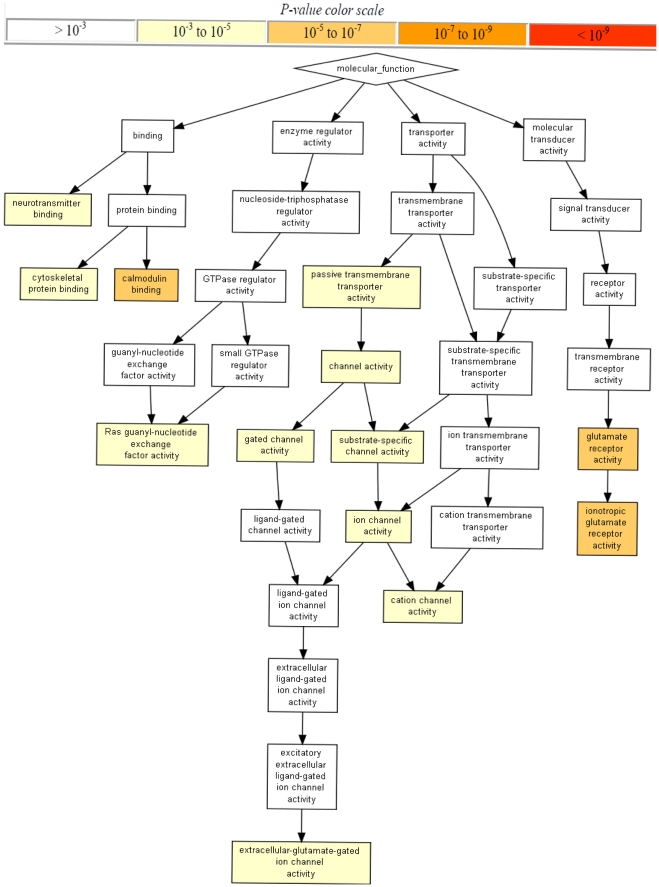
Gene function ontology enrichment by epistatic signals in the MICROS population.

Epistatic genes annotated from the less significant SNP pairs (i.e. −log_10_P_pair_>6.5 and −log_10_P_int_>6.5) in MICROS (910 genes from 1326 pairs) and CROATIAN (984 genes from 1260 pairs) were tested for GO enrichment. The GO terms enriched by epistatic genes in MICROS showed a complicated relationship among biological functions (e.g. calmodulin binding, transporter activity) and highlighted the importance of glutamate receptor activity (Figure 3). Clearly, more than 50% of the GO terms enriched (P<1.0E-05) in MICROS were also enriched in CROATIAN (Table 3, Figures S3 and S4) which suggested GO terms regarding nervous system, synapse, glutamate receptors and plasma membrane were important in both populations. Comparing the epistatic genes enriched the 13 replicated GO terms (Table 3), we found 82 genes shared by both populations including *SLC2A9* and a number of glutamate receptor genes (*GRID1*, *GRIK1*, *GRIK2*, *GRM7* and *GRIN2A*) (Table S2). Surprisingly, 53 out of the 82 shared epistatic genes are previously published GWA loci [31] associated with phenotypes such as multiple sclerosis, Alzheimer's disease, bipolar disorder and schizophrenia, cognition and diabetes. Among SNP pairs where both SNPs were gene annotated and with at least one of the 82 shared epistatic genes from the retained results, we found 49 epistatic gene pairs (correspondingly to 120 SNP pairs) in MICROS were replicated in CROATIAN including *SLC2A9* – *LRRC16A* (interaction between two SUA candidate genes) (Table S3). Two gene pairs were replicated exactly at the SNP level: rs737267 (*SLC2A9*) – rs4085921 (*GPC6*) and rs737267 (*SLC2A9*) – rs2302558 (*CLEC16A*). All the 49 epistatic gene pairs involved *SLC2A9* and of these 5 involved interactions with other shared epistatic genes: *ERC2*, *GPC6*, *CTNND2*, *CNTNAP2* and *PTPRD*. However, most of the 49 replicated gene pairs had a relatively weak interaction signal (−log_10_P_int_<5).

**Table 3:** Gene ontology terms enriched by epistatic genes in the MICROS population and their replication in the CROATIAN population.*

GO Term	Description	MICROS	CROATIAN
GO:0048731^a^	system development	2.88E-10	1.50E-08
GO:0007166^a^	cell surface receptor linked signaling pathway	6.26E-09	*(-)*
GO:0007268^a^	synaptic transmission	1.85E-08	1.68E-05
GO:0007169^a^	transmembrane receptor protein tyrosine kinase signaling pathway	3.27E-07	*(-)*
GO:0030224^a^	monocyte differentiation	1.09E-06	*(-)*
GO:0007611^a^	learning or memory	2.40E-06	*(-)*
GO:0007399^a^	nervous system development	4.53E-06	4.39E-06
GO:0023052^a^	signalling	6.22E-06	5.78E-04
GO:0031644^a^	regulation of neurological system process	6.32E-06	5.63E-04
GO:0050890^a^	cognition	8.41E-06	*(-)*
GO:0007267^a^	cell-cell signalling	9.46E-06	*(-)*
GO:0007165^a^	signal transduction	9.53E-06	6.35E-07
GO:0008066^b^	glutamate receptor activity	3.39E-06	5.35E-05
GO:0004970^b^	ionotropic glutamate receptor activity	7.82E-06	*(-)*
GO:0005516^b^	calmodulin binding	9.68E-06	9.05E-05
GO:0044459^c^	plasma membrane part	1.49E-08	1.38E-07
GO:0030054^c^	cell junction	1.72E-08	2.25E-07
GO:0044456^c^	synapse part	2.11E-07	4.95E-08
GO:0045202^c^	synapse	2.96E-06	3.07E-08
GO:0005886^c^	plasma membrane	7.09E-06	5.28E-07

*: displayed only GO terms with P<1.0E-05; 910 and 984 epistatic genes used in MICROS and CROATIAN respectively; *(-)*: no enrichment at the P<1.0E-03 level.

a : gene process ontology;

b : gene function ontology;

c : gene component ontology.

## Discussion

Using a full pair-wise genome-wide association scan we generated a profile of epistasis in SUA levels in the Italian MICROS population. No epistatic SNP pairs reached the Bonferroni adjusted threshold for the pair-wise genome scan, which was not a surprise because of the relatively small population size. Nonetheless, we found that the *SLC2A9* gene may be an important epistatic locus interacting with multiple loci across the genome more frequently than expected by chance (Tables 2 and S1). Two *SLC2A9* epistatic pairs (*NFIA* – *SLC2A9* and *SLC2A9* – *ESRRAP2*) were significant in SNP-genome scans and jointly explained 8.0% of the phenotypic variance in SUA levels where 3.4% was explained by their interaction components. However, caution should be taken in light of the billions of tests performed, the potential overestimation of the variance explained and the limited replication of the two pairs. The *NFIA* (nuclear factor I/A) gene is known as a cellular transcription DNA replication factor and its haploinsufficiency is associated with a central nervous system malformation syndrome and ureteral and renal defects [32]. Recent GWA studies showed *NFIA* was responsible for celiac disease [33] and ventricular depolarization and conduction [34]. The *ESRRAP2* (estrogen-related receptor alpha pseudogene 2) is not yet known to have any related functions.

The *SLC2A9* gene is known to interact with sex, age and dietary patterns [35], [36], [37] and GWA studies have identified several common variants mapping to various introns and exons of the locus [11], [38], [39], [40]. The protein GLUT9 encoded by *SLC2A9* is a class II glucose/fructose transporter as well as high-capacity/low-affinity urate transporter with two isoforms expressed in basolateral and apical membranes of proximal renal tubular cells respectively [41]. The complexity in the *SLC2A9* polymorphism was demonstrated further in two recent studies linking SUA levels with human cognitive aging [42] and Parkinson's disease [7]. Here we showed that several GWA significant SNPs of *SLC2A9* were involved in epistatic interactions, which adds another dimension of studying the *SLC2A9* polymorphism and provides new clues of the genetic mechanism underlying SUA levels.

The impact of SUA levels on human cognitive dysfunction has been investigated in recent years [7], [42], [43], [44]. It is still unclear though whether high SUA levels causes disease associated aging or vice versa. Considering most SUA associated genes identified from GWA studies encode transporters, it is natural to speculate these transporter genes interact with others that have moderate or low marginal effects (thus not yet discovered in GWA studies) but with certain regulatory roles. Our epistasis results and GO enrichment analyses supported the speculation and showed that numerous *SLC2A9* interacting genes had clear neuronal influence (e.g. synapse, glutamate receptors) and/or membrane functions (Tables 2, S2, S3). It is noteworthy that the GO enrichment results were almost identical after removing *SLC2A9* and genes that interacted with it (8 out of 910 unique genes in MICROS). The epistatic genes encoding glutamate receptors (common in both populations, Table S2) are particularly interesting because glutamate receptors are implicated in the pathologies of a number of neurodegenerative diseases due to their central role in excitotoxicity and their prevalence throughout the central nervous system [45], [46]. A rat study showed that uric acid could protect the neurons from glutamate-induced toxicity [47]. Furthermore, a number of shared epistatic genes in the two populations (Table S2) suggested links between SUA levels and various diseases such as autism (*CNTNAP2*, *POU6F2*, *MYO1D*), schizophrenia and bipolar disorder (*ERC2*, *ROBO1*, *ROBO2*, *CNTNAP2*, *GRM7*, *SYNE1*, *CNTN5*, *ANKS1B*), Alzheimer's disease (*EPHA4*, *CNTN4*, *ADCY8*, *PCSK5*, *CUBN*, *SORCS1*, *RORA*, *GRIN2A*), Parkinson's disease (*DLG2*), sclerosis (*RGS7*, *CNTN4*, *IGF2R*, *SH3GL2*, *GPC5*, *GPC6*, *GRIN2A*, *MYH9*), and diabetes (*CD69*, *PTPRD*, *SORCS1*, *PREX1*).

Statistically detecting and replicating an epistatic pair of SNPs is far more challenging than for a single SNP signal for a number of reasons. Whereas a single SNP association is dependent on strong LD between the marker SNP and causative variant, detection of epistasis requires strong LD for both loci. Small allele frequency changes (e.g. 10%) between detection and replication populations can lead to a dramatic loss of power in replication. Such small allele frequency changes are not uncommon across the small and/or isolated populations. The issue of missing joint genotype class in replication populations (an extreme case of allele frequency change) increases the difficulty of replication, especially when the epistatic pair for test has a rare genotype class (e.g. less than 4 individuals in Table 2). These challenges support the case for exploring epistatic effects and evidence for their replication at the gene and/or pathway levels [48].

The lack of statistical replication at the SNP level may be not too surprising considering that the epistatic signals detected in one isolated sample were to replicate in another isolated sample (CROATIAN) or a less isolated but smaller sample (i.e. SOCCS). Even when all the retained SNP pairs were considered regardless, we just found two SNP pairs (out of 212933) were replicated at the SNP level. When testing for replication at the GO level, we found 13 GO enriched terms (>50%) were replicated. Clearly replication improved dramatically as the level changes from SNP to GO annotation. A high level replication (i.e. gene or GO/pathway) does not guarantee a SNP level replication but is valuable to understand the biology of interest. Therefore, we recommend testing for replication of epistasis signals at all three levels. For example, rs737267 (*SLC2A9*) – rs4085921 (*GPC6*) was replicated at all three levels, rs737267 (*SLC2A9*) – rs2302558 (*CLEC16A*) at the SNP and gene levels but not the GO level as *CLEC16A* is not associated with an enriched GO term. Both *GPC6* (glypican 6) and *CLEC16A* (C-type lectin domain family 16, member A) are associated with multiple sclerosis [49], [50], [51], [52].

In addition to epistasis signal replication, this study also raises a number of issues in GWA epistasis studies. First, it is difficult to detect and replicate genome-wide epistasis signals in small samples because of low power [53] and the problem of missing joint genotype classes (i.e. 26.3% of the total SNP pairs skipped in MICROS). It is suboptimal to ignore SNP pairs with missing genotype classes as some might be true epistatic signals. Methods such as the allelic model used in PLINK [17] and Pseudohaplotype [54] may be helpful to this situation but require further investigation. A large sample size and use of relatively common SNPs (e.g. minor allele frequency >5%) are a partial solution. Second, proper genome-wide thresholds remain to be defined. Bonferroni adjusted thresholds may be appropriate when the total number of tests is close to the effective number of independent tests [55] but may become over stringent when more SNPs are genotyped in GWA studies. Permutation can be a good option but is not yet feasible for GWA epistasis studies due to the excessive computing demand. A set of consensus thresholds [26] will be very useful to guide future GWA epistasis studies. Third, trait normality is critical to the detection of epistasis so as to avoid inflated test statistics simply because more parameters are fitted in an epistatic model than an additive model. Increasing the sample size is a good way to recover the power reduced by the use of quantile normalisation. When it is not possible to do so, a good alternative is to use GO enrichment and pathway analyses [56] to rescue some true signals that are not genome-wide significant but jointly important biologically. It is arguable what threshold should be used to select a proper set of less significant epistatic pairs that includes most true signals and excludes noise as much as possible. A different threshold (other than −log_10_P_int_ of 6.5 used here) could be chosen as long as the big cluster of *SLC2A9* pairs was mostly excluded (Figure 2) and sufficient epistatic genes were available for the GO enrichment analysis. If we considered a threshold of 7.62, there would be only 143 epistatic genes (from 111 SNP pairs) in MICROS and 97 genes (from 96 SNP pairs) in CROATIAN available for the enrichment analyses. The SNP-gene annotation method could affect the GO analysis as well. Here we took a commonly used method based on map distances and allowing only one gene per SNP. This method is generally effective but has a problem when genes overlap or the distances to two genes are equal. In that case, either gene could be quoted depending on which appears first in the annotation results. Such cases were rare so our GO enrichment results were little affected. Nonetheless, new methods are needed to make even more effective use of epistasis signals than our demonstration where epistatic genes were treated independently and more than 40% epistatic SNPs were not gene annotated and hence not considered.

In summary, this study characterized epistasis signals in SUA levels in the MICROS population and shows that *SLC2A9* may be an important epistatic locus interacting with multiple loci across the genome, including those with neuronal influence and/or associating with neurological disorders. Two *SLC2A9* epistatic pairs were genome-wide significant and explained additional phenotypic variance in SUA levels via interactions. We conclude that GWA epistasis study is useful and can provide new insights into the trait of interest in conjunction with GO/pathway enrichment analysis utilising less significant epistatic signals.

## Supporting Information

Figure S1 **The QQ plots for single-SNP based genome-wide association scans in the MICROS and CROATIAN populations.**
(TIF)

Figure S2 **The joint genotype – phenotype maps for two genome-wide significant epistatic pairs.**
(TIF)

Figure S3 **Gene component ontology terms enriched by epistatic genes in the MICROS population.**
(TIF)

Figure S4 **Gene component ontology terms enriched by epistatic genes in the CROATIAN populations.**
(TIF)

Table S1 ***SLC2A9*** **involved epistatic pairs (−log_10_P_pair_>6.5 and −log_10_P_int_>6.5) in MICROS.**
(PDF)

Table S2 **Epistatic genes in the replicated GO terms shared by the MICROS and CROATIAN populations.**
(PDF)

Table S3 **Epistatic pairs with at least one shared GO gene and replicated in MICROS and CROATIAN.**
(PDF)
