# Supplementary figures and images for: Characterisation of Genome-Wide Association Epistasis Signals for Serum Uric Acid in Human Population Isolates
Source: PLoS One. 2011 Aug 19;6(8):e23836. doi: 10.1371/journal.pone.0023836 (PMC3158795; doi:10.1371/journal.pone.0023836)

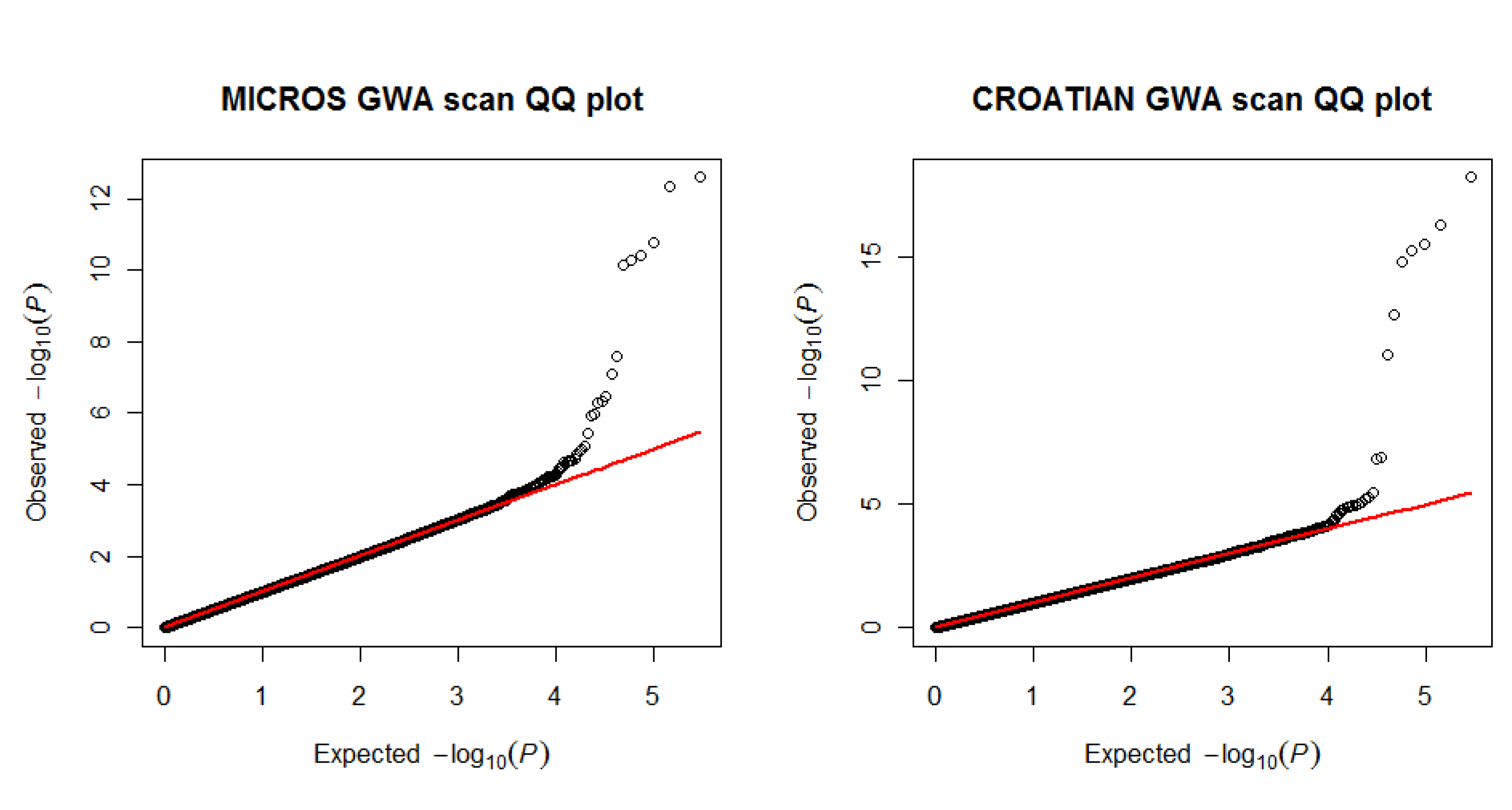

Supplement: Figure S1 — The QQ plots for single-SNP based genome-wide association scans in the MICROS and CROATIAN populations. (TIF) [file pone.0023836.s001.tif]

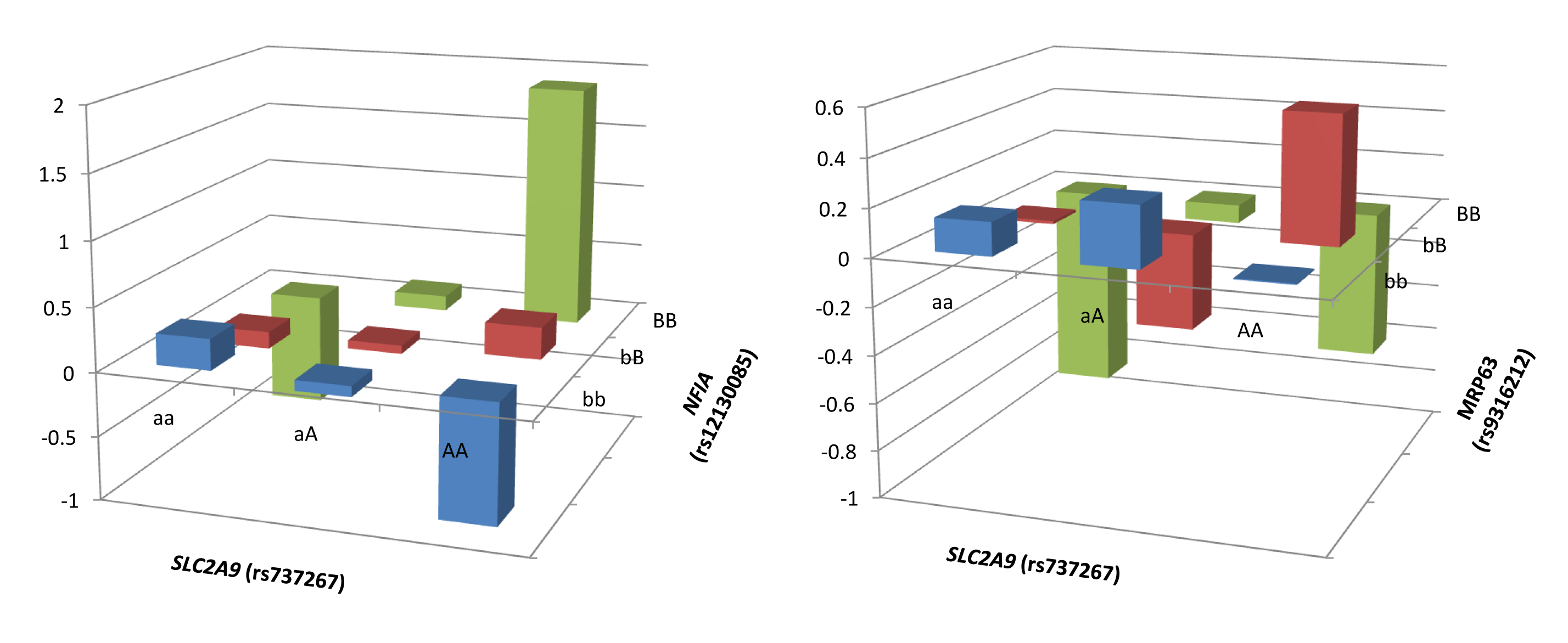

Supplement: Figure S2 — The joint genotype – phenotype maps for two genome-wide significant epistatic pairs. (TIF) [file pone.0023836.s002.tif]

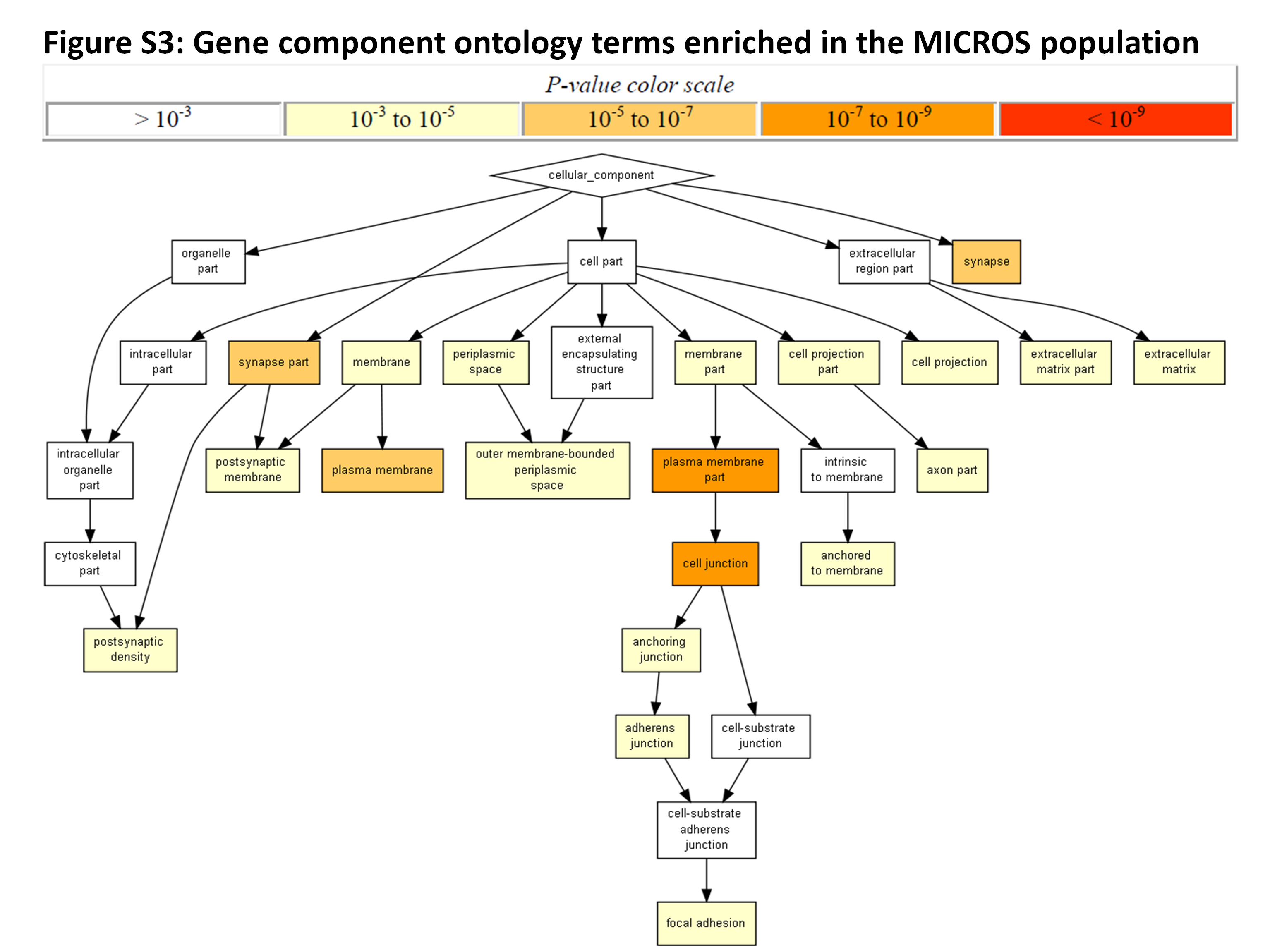

Supplement: Figure S3 — Gene component ontology terms enriched by epistatic genes in the MICROS population. (TIF) [file pone.0023836.s003.tif]

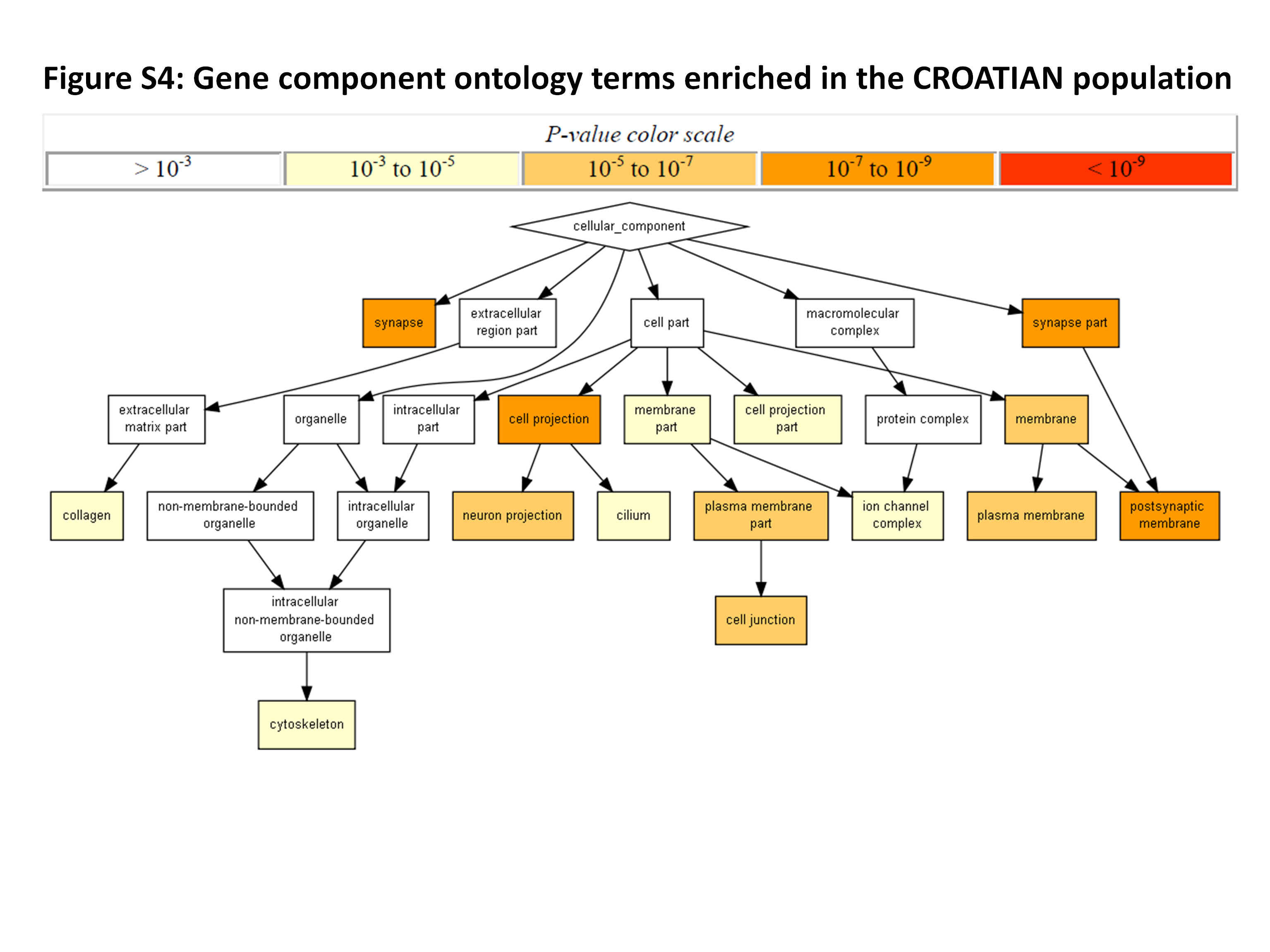

Supplement: Figure S4 — Gene component ontology terms enriched by epistatic genes in the CROATIAN populations. (TIF) [file pone.0023836.s004.tif]
