# Supplementary material for: Characterisation of Genome-Wide Association Epistasis Signals for Serum Uric Acid in Human Population Isolates
Source: PLoS One. 2011 Aug 19;6(8):e23836. doi: 10.1371/journal.pone.0023836 (PMC3158795; doi:10.1371/journal.pone.0023836)
Supplement: Table S1 — SLC2A9 involved epistatic pairs (−log10Ppair>6.5 and −log10Pint>6.5) in MICROS. (PDF) [file pone.0023836.s005.pdf]

Table S1: *SLC2A9* involved epistatic pairs ($-\log_{10}P_{pair} > 6.5$ and $-\log_{10}P_{int} > 6.5$) in MICROS

**SNP_1**	**chr_1**	**$gene_1$**	**SNP_2**	**chr_2**	**$gene_2$**	**P_{pair}**	**P_{int}**	**MGC**
rs12130085	1	*NFIA*	rs13129697	4	*SLC2A9*	13.86	6.54	2
rs12130085	1	*NFIA*	rs737267	4	*SLC2A9*	16.38	8.2	2
rs12130085	1	*NFIA*	rs13131257	4	*SLC2A9*	13.8	6.75	2
rs1464258	4	*SLC2A9*	rs838795	5	*C5orf50*	7.04	7.08	17
rs733175	4	*SLC2A9*	rs1173723	5	*(-)*	10.45	6.8	6
rs733175	4	*SLC2A9*	rs1818116	5	*(-)*	11.43	7.27	7
rs4697895	4	*SLC2A9*	rs4747887	10	*PFKFB3*	7.16	6.59	15
rs4697895	4	*SLC2A9*	rs7099083	10	*PFKFB3*	7.35	6.78	15
rs1107710	4	*SLC2A9*	rs7099083	10	*PFKFB3*	6.66	6.78	11
rs1107710	4	*SLC2A9*	rs12281918	11	*FLI1*	7.66	6.63	2
rs4697902	4	*SLC2A9*	rs2374532	12	*(-)*	7.16	6.56	1
rs4697895	4	*SLC2A9*	rs1887589	13	*(-)*	8.45	7.33	2
rs4697902	4	*SLC2A9*	rs1887589	13	*(-)*	7.39	6.67	2
rs737267	4	*SLC2A9*	rs9316212	13	*ESRRAP2*	16.21	7.97	2
rs13131257	4	*SLC2A9*	rs9316212	13	*ESRRAP2*	14.98	7.88	2
rs2867394	4	*SLC2A9*	rs7141105	14	*SLC8A3*	8.06	6.58	4
rs733175	4	*SLC2A9*	rs137180	22	*SEZ6L*	10.09	6.58	4

SNP_1 (SNP_2): the first (second) SNP name;

chr_1 (chr_2): the chromosome where SNP_1 (SNP_2) locates

$gene_1$ ($gene_2$): symbol of the gene annotated by SNP_1 (SNP_2);

P_{pair}: $-\log_{10}$ P value of the whole pair test;

$P_{int:}$ $-\log_{10}$ P value of the interaction test;

MGC: count of number of individuals in the minor joint genotype class

(-) : no gene annotated
