# Supplementary material for: Characterisation of Genome-Wide Association Epistasis Signals for Serum Uric Acid in Human Population Isolates
Source: PLoS One. 2011 Aug 19;6(8):e23836. doi: 10.1371/journal.pone.0023836 (PMC3158795; doi:10.1371/journal.pone.0023836)
Supplement: Table S2 — Epistatic genes in the replicated GO terms shared by the MICROS and CROATIAN populations. (PDF) [file pone.0023836.s006.pdf]

Table S2: Epistatic genes in the replicated GO terms shared by the MICROS and CROATIAN populations

Symbol	Region	Gene Name	GWA information and disease associated*
PLXNA2	1q32.2	plexin a2	Cardiac hypertrophy
RGS7	1q23.1	regulator of g-protein signaling 7	Multiple sclerosis
UBR4	1p36.13	ubiquitin protein ligase e3 component n-recognin 4	*(-)*
EPHA4	2q36.1	eph receptor a4	Alzheimer's Disease, mild cognitive impairment
GLI2	2q14.2	gli family zinc finger 2	Erectile dysfunction and prostate cancer treatment
PARD3B	2q33.3	par-3 partitioning defective 3 homolog b (c. elegans)	Knee osteoarthritis
SLC8A1	2p23-p22	solute carrier family 8 (sodium/calcium exchanger), member 1	*(-)*
CHL1	3p26.3	cell adhesion molecule with homology to l1cam (close homolog of l1)	Scoliosis; lipid-lowering response to statins
CNTN4	3p26.3	contactin 4	Alzheimer's disease; Amyotrophic lateral sclerosis; Blood pressure; Acenocoumarol maintenance dosage
ERC2	3p14.3	elks/rab6-interacting/cast family member 2	Bipolar disorder and schizophrenia; Hip geometry
GRM7	3p26.1	glutamate receptor, metabotropic 7	Neuroticism Anxiety; Bipolar disorder; Major depressive disorder; Panic disorder
HRH1	3p25	histamine receptor h1	*(-)*
PLCH1	3q25.31	phospholipase c, eta 1	*(-)*
ROBO1	3p12.3	roundabout, axon guidance receptor, homolog 1 (drosophila)	Brain imaging in Schizophrenia
ROBO2	3p12.3	roundabout, axon guidance receptor, homolog 2 (drosophila)	Brain imaging in Schizophrenia
SRGAP3	3p25.3	slit-robo rho gtpase activating protein 3	*(-)*
ARHGAP24	4q21.23	rho gtpase activating protein 24	Electrocardiographic traits
DCHS2	4q31.3	dachsous 2 (drosophila)	*(-)*
KCNIP4	4p15.2	kv channel interacting protein 4	Suicidal ideation
SLC2A9	4p16.1	solute carrier family 2 (facilitated glucose transporter), member 9	Serum urate; Serum uric acid
CAMK4	5q22.1	calcium/calmodulin-dependent protein kinase iv	blood pressure and arterial stiffness
CDH12	5p14.3	cadherin 12, type 2 (n-cadherin 2)	Waist circumference
CTNND2	5p15.2	catenin (cadherin-associated protein), delta 2 (neural plakophilin-related arm-repeat protein)	Myopia
ENC1	5q13	ectodermal-neural cortex 1 (with btb-like domain)	*(-)*
ODZ2	5q34	odz, odd oz/ten-m homolog 2 (drosophila)	*(-)*
BAI3	6q13	brain-specific angiogenesis inhibitor 3	Lipid-lowering response to statins
GRIK2	6q16.3	glutamate receptor, ionotropic, kainate 2	Cardiac hypertrophy; Biochemical measures
IGF2R	6q25.3	insulin-like growth factor 2 receptor	Multiple sclerosis
NEDD9	6p25-p24	neural precursor cell expressed, developmentally down-regulated 9	*(-)*
NKAIN2	6q22.31	na+/k+ transporting atpase interacting 2	Neuroticism
RPS6KA2	6q27	ribosomal protein s6 kinase, 90kda, polypeptide 2	*(-)*
SYNE1	6q25.2	spectrin repeat containing, nuclear envelope 1	Bipolar disorder and major depressive disorder; blood pressure and arterial stiffness
UNC93A	6q27	unc-93 homolog a (c. elegans)	*(-)*
CHRM2	7q31-q35	cholinergic receptor, muscarinic 2	*(-)*
CNTNAP2	7q35	contactin associated protein-like 2	Bipolar disorder and schizophrenia; Bone mineral density; Autism
POU6F2	7p14.1	pou class 6 homeobox 2	Autism
ADCY8	8q24.22	adenylate cyclase 8 (brain)	Alzheimer's disease

DLC1	8p22	deleted in liver cancer 1	*(-)*
NRG1	8p12	neuregulin 1	Hirschsprung's disease; Hip geometry
DIRAS2	9q22.2	diras family, gtp-binding ras-like 2	Cognitive test performance; Protein quantitative trait loci
GNA14	9q21	guanine nucleotide binding protein (g protein), alpha 14	*(-)*
LPPR1	9q31.1	lipid phosphate phosphatase-related protein type 1	*(-)*
NTRK2	9q22.1	neurotrophic tyrosine kinase, receptor, type 2	*(-)*
PCSK5	9q21.13	proprotein convertase subtilisin/kexin type 5	Alzheimer's disease
PTPRD	9p24.1	protein tyrosine phosphatase, receptor type, d	Type 2 diabetes; Platelet aggregation; Partial epilepsies; Restless legs syndrome
SH3GL2	9p22.2	sh3-domain grb2-like 2	Cognitive performance; Heart failure; Multiple sclerosis
COL13A1	10q22.1	collagen, type xiii, alpha 1	Non-alcoholic fatty liver disease
CUBN	10p13	cubilin (intrinsic factor-cobalamin receptor)	Urinary albumin excretion; Alzheimer's disease; Folate pathway vitamin levels
DNMBP	10q24.2	dynamin binding protein	*(-)*
DOCK1	10q26.13	dedicator of cytokinesis 1	*(-)*
GRID1	10q23.1	glutamate receptor, ionotropic, delta 1	Cardiac structure and function
PRKG1	10q11.2	protein kinase, cgmp-dependent, type i	*(-)*
SORCS1	10q23-q25	sortilin-related vps10 domain containing receptor 1	Diabetes; Alzheimer's disease;
CNTN5	11q22.1	contactin 5	Bipolar disorder and schizophrenia; Atrial fibrillation; brain aging
DLG2	11q14.1	discs, large homolog 2 (drosophila)	Parkinson's disease; Protein quantiative trait loci
KCNJ5	11q24	potassium inwardly-rectifying channel, subfamily j, member 5	*(-)*
KIRREL3	11q24.2	kin of irre like 3 (drosophila)	Response to antipsychotic treatment; Attention deficit hyperactivity disorder and conduct disorder;
OPCML	11q25	opioid binding protein/cell adhesion molecule-like	*(-)*
ZBTB16	11q23.2	zinc finger and btb domain containing 16	Conduct disorder
ANKS1B	12q23.1	ankyrin repeat and sterile alpha motif domain containing 1b	Schizophrenia; body mass index; waist circumference
CD69	12p13.31	cd69 molecule	Type 1 diabetes
NINJ2	12p13.33	ninjurin 2	Stroke
PDE3A	12p12.2	phosphodiesterase 3a, cgmp-inhibited	Cardiovascular disease; Male infertility; Height
GPC5	13q31.3	glypican 5	Multiple sclerosis; Serum metabolites; Height; Lung cancer;
GPC6	13q31.3	glypican 6	Neuroticism; Attention deficit hyperactivity disorder; Tonometry; Kidney aging
MCF2L	13q34	mcf.2 cell line derived transforming sequence-like	Hemostatic factors for cardiovascular disease
PCDH9	13q21.32	protocadherin 9	*(-)*
PTGER2	14q22	prostaglandin e receptor 2 (subtype ep2), 53kda	*(-)*
RORA	15q22.2	rar-related orphan receptor a	Cardiovascular disease; Alzheimer's disease; Age at menarche; Asthma; Depression
GRIN2A	16p13.2	glutamate receptor, ionotropic, n-methyl d-aspartate 2a	Smoking; Alzheimer's disease; Amyotrophic lateral sclerosis
MYO1D	17q11.2	myosin id	Pancreatic cancer; Autism
PRKCA	17q24.2	protein kinase c, alpha	Cardiac ventricular conduction
MBP	18q23	myelin basic protein	*(-)*
RNF138	18q12.1	ring finger protein 138	*(-)*
CACNA1A	19p13	calcium channel, voltage-dependent, p/q type, alpha 1a subunit	*(-)*
MED16	19p13.3	mediator complex subunit 16	*(-)*
PLCB1	20p12.3	phospholipase c, beta 1 (phosphoinositide-specific)	Cognitive test performance
PREX1	20q13.13	phosphatidylinositol-3,4,5-trisphosphate-dependent rac exchange factor 1	Blood pressure and arterial stiffness; Type 2 diabetes

RSPO4	20p13	r-spondin family, member 4	*(-)*
AGPAT3	21q22.3	1-acylglycerol-3-phosphate o-acyltransferase 3	*(-)*
GRIK1	21q21.3	glutamate receptor, ionotropic, kainate 1	Lipid-lowering response to statins; Hyperactive-impulsive symptoms; Breast cancer
MYH9	22q12.3	myosin, heavy chain 9, non-muscle	Glomerulosclerosis; End-stage renal disease; Optic disc size

(-): no information

*: GWA information extracted from the NHGRI GWAS Catalog (http://www.genome.gov/gwastudies/)
