# Supplementary material for: Characterisation of Genome-Wide Association Epistasis Signals for Serum Uric Acid in Human Population Isolates
Source: PLoS One. 2011 Aug 19;6(8):e23836. doi: 10.1371/journal.pone.0023836 (PMC3158795; doi:10.1371/journal.pone.0023836)
Supplement: Table S3 — Epistatic pairs with at least one shared GO gene and replicated in MICROS and CROATIAN. (PDF) [file pone.0023836.s007.pdf]

Table S3: Epistatic pairs with at least one shared GO gene and replicated in MICROS and CROATIAN

SNP_1	chr_1	$gene_1$	SNP_2	chr_2	$gene_2$	P_{pair}	P_{int}	MGC	population
rs3767511	1	*CACNA1S*	rs6449213	4	*SLC2A9*	10.93	3.95	1	MICROS
rs3767511	1	*CACNA1S*	rs1014290	4	*SLC2A9*	10.14	3.58	1	MICROS
rs4915215	1	*CACNA1S*	rs1014290	4	*SLC2A9*	15.93	3.51	23	CROATIAN
rs12566907	1	*DPYD*	rs733175	4	*SLC2A9*	8.34	3.93	4	MICROS
rs7548189	1	*DPYD*	rs737267	4	*SLC2A9*	12.09	3.28	5	MICROS
rs7548189	1	*DPYD*	rs13131257	4	*SLC2A9*	10.87	3.39	4	MICROS
rs10875051	1	*DPYD*	rs4505821	4	*SLC2A9*	7.21	3.97	4	CROATIAN
rs12041436	1	*DPYD*	rs4505821	4	*SLC2A9*	8.16	4.63	4	CROATIAN
rs4415625	1	*DPYD*	rs4505821	4	*SLC2A9*	8.08	4.5	4	CROATIAN
rs4415625	1	*DPYD*	rs6820230	4	*SLC2A9*	7.02	5.74	4	CROATIAN
rs827497	1	*DPYD*	rs10805346	4	*SLC2A9*	12.52	4.04	32	CROATIAN
rs7559750	2	*DOCK10*	rs6449213	4	*SLC2A9*	10.68	3.55	2	MICROS
rs6436516	2	*DOCK10*	rs6449213	4	*SLC2A9*	10.65	3.56	2	MICROS
rs921281	2	*DOCK10*	rs10805346	4	*SLC2A9*	12.35	3.38	64	CROATIAN
rs2303325	2	*GALNT14*	rs6449213	4	*SLC2A9*	10.59	3.51	1	MICROS
rs12621279	2	*GALNT14*	rs13131257	4	*SLC2A9*	13.94	3.55	13	CROATIAN
rs720755	2	*LRP1B*	rs737267	4	*SLC2A9*	12.05	3.56	7	MICROS
rs720755	2	*LRP1B*	rs13131257	4	*SLC2A9*	11.14	3.76	5	MICROS
rs1949871	2	*LRP1B*	rs1014290	4	*SLC2A9*	15.89	3.46	28	CROATIAN
rs1880620	2	*LRP1B*	rs1014290	4	*SLC2A9*	15.87	3.48	31	CROATIAN
rs10490229	2	*NRXN1*	rs737267	4	*SLC2A9*	12.41	3.98	4	MICROS
rs9750635	2	*NRXN1*	rs13131257	4	*SLC2A9*	14.05	3.58	4	CROATIAN
rs10185359	2	*ZEB2*	rs10805346	4	*SLC2A9*	8.92	3.3	8	MICROS
rs7564469	2	*ZEB2*	rs733175	4	*SLC2A9*	10.97	3.3	4	CROATIAN
rs2318154	3	*EPHA6*	rs13131257	4	*SLC2A9*	10.12	3.27	1	MICROS
rs1875281	3	*EPHA6*	rs10805346	4	*SLC2A9*	11.58	3.51	17	CROATIAN
rs17711589	3	*ERC2*	rs4447863	4	*SLC2A9*	7.13	4.51	19	MICROS
rs815470	3	*ERC2*	rs1014290	4	*SLC2A9*	16.89	3.63	4	CROATIAN
rs1524966	3	*MAGI1*	rs733175	4	*SLC2A9*	7.16	3.33	2	MICROS
rs1864509	3	*MAGI1*	rs733175	4	*SLC2A9*	7.56	3.82	9	MICROS
rs1430416	3	*MAGI1*	rs733175	4	*SLC2A9*	7.3	3.39	8	MICROS
rs6778911	3	*MAGI1*	rs13129697	4	*SLC2A9*	16.3	3.53	4	CROATIAN
rs737267	4	*SLC2A9*	rs10016365	4	*ANK2*	13.11	4.29	3	MICROS
rs733175	4	*SLC2A9*	rs1385662	4	*ANK2*	12.14	3.57	11	CROATIAN
rs10805346	4	*SLC2A9*	rs17013734	4	*PHF17*	8.98	3.72	8	MICROS
rs6449213	4	*SLC2A9*	rs11933240	4	*PHF17*	14.94	3.35	7	CROATIAN
rs13131257	4	*SLC2A9*	rs1501776	5	*CTNND2*	11.19	4.24	15	MICROS
rs13129697	4	*SLC2A9*	rs881675	5	*CTNND2*	16.6	3.38	7	CROATIAN
rs13129697	4	*SLC2A9*	rs26478	5	*CTNND2*	17.23	3.4	11	CROATIAN
rs1014290	4	*SLC2A9*	rs881675	5	*CTNND2*	17.03	4.44	4	CROATIAN
rs733175	4	*SLC2A9*	rs881675	5	*CTNND2*	11.52	3.81	4	CROATIAN
rs13129697	4	*SLC2A9*	rs7718587	5	*PPP2R2B*	10.89	3.55	9	MICROS
rs6449213	4	*SLC2A9*	rs974097	5	*PPP2R2B*	11.75	3.99	1	MICROS
rs6449213	4	*SLC2A9*	rs1663333	5	*PPP2R2B*	11.08	3.44	1	MICROS
rs13131257	4	*SLC2A9*	rs161039	5	*PPP2R2B*	14.19	3.41	6	CROATIAN
rs6449213	4	*SLC2A9*	rs161039	5	*PPP2R2B*	15.59	3.52	6	CROATIAN
rs1014290	4	*SLC2A9*	rs161039	5	*PPP2R2B*	17.26	4.46	11	CROATIAN

rs733175	4	*SLC2A9*	rs161039	5	*PPP2R2B*	12.72	4.76	8 CROATIAN
rs733175	4	*SLC2A9*	rs11748210	5	*PPP2R2B*	10.74	3.61	20 CROATIAN
rs733175	4	*SLC2A9*	rs1368379	5	*PPP2R2B*	12	3.89	1 CROATIAN
rs10805346	4	*SLC2A9*	rs4499954	6	*ARHGAP18*	9.15	3.34	12 MICROS
rs10805346	4	*SLC2A9*	rs9321174	6	*ARHGAP18*	11.87	3.74	53 CROATIAN
rs13129697	4	*SLC2A9*	rs1980450	6	*LRRC16A*	10.72	3.49	23 MICROS
rs6449213	4	*SLC2A9*	rs10498725	6	*LRRC16A*	11.32	3.29	1 MICROS
rs1014290	4	*SLC2A9*	rs7748771	6	*LRRC16A*	10.68	3.6	1 MICROS
rs1014290	4	*SLC2A9*	rs1980450	6	*LRRC16A*	11.01	4.48	8 MICROS
rs733175	4	*SLC2A9*	rs7748771	6	*LRRC16A*	7.45	3.32	1 MICROS
rs10805346	4	*SLC2A9*	rs441460	6	*LRRC16A*	11.86	3.52	71 CROATIAN
rs737267	4	*SLC2A9*	rs1105953	7	*AUTS2*	11.76	3.61	18 MICROS
rs10805346	4	*SLC2A9*	rs2293496	7	*AUTS2*	13.41	4.4	10 CROATIAN
rs13129697	4	*SLC2A9*	rs2293496	7	*AUTS2*	17.29	3.77	9 CROATIAN
rs13131257	4	*SLC2A9*	rs2237526	7	*CACNA2D1*	10.92	3.62	4 MICROS
rs1014290	4	*SLC2A9*	rs2237526	7	*CACNA2D1*	10.74	3.58	3 MICROS
rs733175	4	*SLC2A9*	rs2299178	7	*CACNA2D1*	7.25	3.27	3 MICROS
rs733175	4	*SLC2A9*	rs2237526	7	*CACNA2D1*	7.53	3.38	3 MICROS
rs10805346	4	*SLC2A9*	rs258684	7	*CACNA2D1*	11.87	3.41	71 CROATIAN
rs13131257	4	*SLC2A9*	rs7809670	7	*CNTNAP2*	11.89	3.3	1 MICROS
rs13129697	4	*SLC2A9*	rs2906300	7	*CNTNAP2*	17.28	3.9	25 CROATIAN
rs10805346	4	*SLC2A9*	rs160359	7	*CREB5*	9.12	3.93	2 MICROS
rs737267	4	*SLC2A9*	rs849322	7	*CREB5*	14.6	3.3	40 CROATIAN
rs10805346	4	*SLC2A9*	rs17134567	7	*SDK1*	8.77	3.4	26 MICROS
rs13129697	4	*SLC2A9*	rs4723454	7	*SDK1*	12.7	4.76	13 MICROS
rs13129697	4	*SLC2A9*	rs17134567	7	*SDK1*	10.83	3.29	10 MICROS
rs737267	4	*SLC2A9*	rs4723454	7	*SDK1*	13.18	4.28	8 MICROS
rs737267	4	*SLC2A9*	rs17134567	7	*SDK1*	12.41	3.99	7 MICROS
rs13131257	4	*SLC2A9*	rs17134567	7	*SDK1*	10.74	3.51	5 MICROS
rs13129697	4	*SLC2A9*	rs17330921	7	*SDK1*	17.22	3.96	9 CROATIAN
rs6449213	4	*SLC2A9*	rs6979125	7	*THSD7A*	10.41	3.45	1 MICROS
rs13131257	4	*SLC2A9*	rs6959645	7	*THSD7A*	17.7	3.59	20 CROATIAN
rs13131257	4	*SLC2A9*	rs7802386	7	*ZNF804B*	10.96	3.96	3 MICROS
rs10805346	4	*SLC2A9*	rs1990020	7	*ZNF804B*	11.67	3.4	26 CROATIAN
rs13131257	4	*SLC2A9*	rs1809437	8	*CPA6*	10.68	3.76	3 MICROS
rs4505821	4	*SLC2A9*	rs2128105	8	*CPA6*	7	3.78	9 CROATIAN
rs10805346	4	*SLC2A9*	rs2725043	8	*CSMD1*	10.03	4.32	28 MICROS
rs13129697	4	*SLC2A9*	rs11783144	8	*CSMD1*	11.44	3.71	8 MICROS
rs737267	4	*SLC2A9*	rs11783144	8	*CSMD1*	11.91	3.3	3 MICROS
rs10805346	4	*SLC2A9*	rs17066956	8	*CSMD1*	12.44	3.61	4 CROATIAN
rs13129697	4	*SLC2A9*	rs2623633	8	*CSMD1*	16.35	3.29	23 CROATIAN
rs737267	4	*SLC2A9*	rs11987743	8	*CSMD1*	15.55	3.35	20 CROATIAN
rs737267	4	*SLC2A9*	rs2623633	8	*CSMD1*	15.5	4.4	13 CROATIAN
rs6449213	4	*SLC2A9*	rs10095133	8	*CSMD1*	15.08	3.36	4 CROATIAN
rs1014290	4	*SLC2A9*	rs6993971	8	*SAMD12*	9.85	3.32	13 MICROS
rs733175	4	*SLC2A9*	rs6993971	8	*SAMD12*	8.42	4.8	8 MICROS
rs13129697	4	*SLC2A9*	rs2450206	8	*SAMD12*	17.06	3.98	9 CROATIAN
rs1014290	4	*SLC2A9*	rs2450206	8	*SAMD12*	15.99	3.61	5 CROATIAN
rs733175	4	*SLC2A9*	rs2450206	8	*SAMD12*	10.82	3.29	5 CROATIAN
rs6449213	4	*SLC2A9*	rs4465021	9	*KDM4C*	10.8	3.72	1 MICROS

rs737267	4	*SLC2A9*	rs17456897	9	*KDM4C*	14.37	3.3	5 CROATIAN
rs13131257	4	*SLC2A9*	rs668205	9	*PTPRD*	11.47	3.71	19 MICROS
rs1014290	4	*SLC2A9*	rs10491914	9	*PTPRD*	9.55	3.28	9 MICROS
rs1014290	4	*SLC2A9*	rs635580	9	*PTPRD*	15.79	3.33	9 CROATIAN
rs733175	4	*SLC2A9*	rs635580	9	*PTPRD*	11.29	3.69	4 CROATIAN
rs13129697	4	*SLC2A9*	rs4750440	10	*FRMD4A*	11.26	3.72	17 MICROS
rs733175	4	*SLC2A9*	rs2446581	10	*FRMD4A*	7.05	3.4	1 MICROS
rs737267	4	*SLC2A9*	rs1327000	10	*FRMD4A*	14.76	3.63	23 CROATIAN
rs733175	4	*SLC2A9*	rs10793040	11	*ARAP1*	7.1	3.37	5 MICROS
rs733175	4	*SLC2A9*	rs10793039	11	*ARAP1*	11.12	3.41	19 CROATIAN
rs2867394	4	*SLC2A9*	rs12281918	11	*FLI1*	7.62	6.02	2 MICROS
rs1107710	4	*SLC2A9*	rs12281918	11	*FLI1*	7.66	6.63	2 MICROS
rs737267	4	*SLC2A9*	rs12281918	11	*FLI1*	12.83	3.43	1 MICROS
rs13129697	4	*SLC2A9*	rs2284786	11	*FLI1*	17.82	3.34	34 CROATIAN
rs737267	4	*SLC2A9*	rs2284786	11	*FLI1*	15.68	3.43	24 CROATIAN
rs6449213	4	*SLC2A9*	rs7971370	12	*GRIP1*	10.98	3.35	1 MICROS
rs4505821	4	*SLC2A9*	rs4486703	12	*GRIP1*	7.92	3.6	15 CROATIAN
rs733175	4	*SLC2A9*	rs9556886	13	*FARP1*	7.08	3.44	5 MICROS
rs10805346	4	*SLC2A9*	rs2390038	13	*FARP1*	12.49	4.21	77 CROATIAN
rs13129697	4	*SLC2A9*	rs536863	13	*FGF14*	10.71	3.51	18 MICROS
rs737267	4	*SLC2A9*	rs4335654	13	*FGF14*	11.79	3.3	1 MICROS
rs13131257	4	*SLC2A9*	rs7491329	13	*FGF14*	11.07	4.13	1 MICROS
rs13129697	4	*SLC2A9*	rs649589	13	*FGF14*	18.08	4.89	7 CROATIAN
rs13129697	4	*SLC2A9*	rs784205	13	*FGF14*	18.07	4.88	7 CROATIAN
rs13129697	4	*SLC2A9*	rs1336703	13	*FGF14*	18.42	5.29	7 CROATIAN
rs13129697	4	*SLC2A9*	rs1415053	13	*FGF14*	16.3	3.38	9 CROATIAN
rs13129697	4	*SLC2A9*	rs7985526	13	*FGF14*	16.44	3.41	9 CROATIAN
rs13129697	4	*SLC2A9*	rs1928510	13	*FGF14*	16.61	3.56	9 CROATIAN
rs737267	4	*SLC2A9*	rs649589	13	*FGF14*	14.68	3.52	5 CROATIAN
rs737267	4	*SLC2A9*	rs784205	13	*FGF14*	14.66	3.51	5 CROATIAN
rs13131257	4	*SLC2A9*	rs649589	13	*FGF14*	14.48	4.08	4 CROATIAN
rs13131257	4	*SLC2A9*	rs784205	13	*FGF14*	14.46	4.08	4 CROATIAN
rs13131257	4	*SLC2A9*	rs1336714	13	*FGF14*	15.19	3.79	10 CROATIAN
rs6449213	4	*SLC2A9*	rs649589	13	*FGF14*	16.37	4.66	4 CROATIAN
rs6449213	4	*SLC2A9*	rs784205	13	*FGF14*	16.35	4.66	4 CROATIAN
rs1014290	4	*SLC2A9*	rs649589	13	*FGF14*	18.21	5.7	5 CROATIAN
rs1014290	4	*SLC2A9*	rs784205	13	*FGF14*	18.19	5.7	5 CROATIAN
rs1014290	4	*SLC2A9*	rs1336703	13	*FGF14*	16.64	4.19	6 CROATIAN
rs737267	4	*SLC2A9*	rs4085921	13	*GPC6*	11.66	3.58	10 MICROS
rs733175	4	*SLC2A9*	rs7321299	13	*GPC6*	7.3	3.33	9 MICROS
rs13129697	4	*SLC2A9*	rs10508009	13	*GPC6*	16.55	3.39	20 CROATIAN
rs737267	4	*SLC2A9*	rs1947078	13	*GPC6*	15.1	3.88	25 CROATIAN
rs737267	4	*SLC2A9*	rs7997642	13	*GPC6*	15.02	3.65	19 CROATIAN
rs737267	4	*SLC2A9*	rs4085921	13	*GPC6*	15.02	3.99	14 CROATIAN
rs737267	4	*SLC2A9*	rs10508009	13	*GPC6*	15.22	4.2	12 CROATIAN
rs737267	4	*SLC2A9*	rs9524163	13	*GPC6*	14.96	3.98	13 CROATIAN
rs737267	4	*SLC2A9*	rs4773754	13	*GPC6*	15.34	4.18	14 CROATIAN
rs737267	4	*SLC2A9*	rs9524175	13	*GPC6*	15.62	4.35	11 CROATIAN
rs733175	4	*SLC2A9*	rs1326132	13	*MIPEP*	7.26	3.36	2 MICROS
rs13131257	4	*SLC2A9*	rs1536299	13	*MIPEP*	14.25	3.56	7 CROATIAN

rs733175	4	*SLC2A9*	rs1861087	14	*FBLN5*	7.5	3.56	2	MICROS
rs13131257	4	*SLC2A9*	rs2246416	14	*FBLN5*	13.63	3.36	17	CROATIAN
rs733175	4	*SLC2A9*	rs7162000	15	*SCG5*	7.59	3.48	3	MICROS
rs10805346	4	*SLC2A9*	rs1406387	15	*SCG5*	11.96	3.44	60	CROATIAN
rs1014290	4	*SLC2A9*	rs3859084	16	*CDH13*	10.26	3.41	8	MICROS
rs13129697	4	*SLC2A9*	rs10514554	16	*CDH13*	17.31	3.72	1	CROATIAN
rs13129697	4	*SLC2A9*	rs4421965	16	*CDH13*	16.75	3.54	23	CROATIAN
rs737267	4	*SLC2A9*	rs10514554	16	*CDH13*	14.91	3.47	1	CROATIAN
rs13131257	4	*SLC2A9*	rs1870846	16	*CDH13*	13.64	3.81	1	CROATIAN
rs13131257	4	*SLC2A9*	rs10514554	16	*CDH13*	14.28	3.53	1	CROATIAN
rs13131257	4	*SLC2A9*	rs8055389	16	*CDH13*	14.48	3.87	2	CROATIAN
rs1014290	4	*SLC2A9*	rs7499717	16	*CDH13*	16.33	3.41	4	CROATIAN
rs13129697	4	*SLC2A9*	rs8059260	16	*CLEC16A*	10.75	3.28	1	MICROS
rs13129697	4	*SLC2A9*	rs2302558	16	*CLEC16A*	10.84	3.44	1	MICROS
rs13129697	4	*SLC2A9*	rs7197758	16	*CLEC16A*	11.01	3.28	1	MICROS
rs737267	4	*SLC2A9*	rs2302558	16	*CLEC16A*	11.66	3.44	1	MICROS
rs13131257	4	*SLC2A9*	rs2302558	16	*CLEC16A*	10.56	3.49	1	MICROS
rs6449213	4	*SLC2A9*	rs8059260	16	*CLEC16A*	11.17	3.83	1	MICROS
rs6449213	4	*SLC2A9*	rs2302558	16	*CLEC16A*	11.25	3.94	1	MICROS
rs6449213	4	*SLC2A9*	rs8055893	16	*CLEC16A*	10.95	3.61	1	MICROS
rs1014290	4	*SLC2A9*	rs8059260	16	*CLEC16A*	10.23	3.33	1	MICROS
rs1014290	4	*SLC2A9*	rs2302558	16	*CLEC16A*	10.44	3.57	1	MICROS
rs1014290	4	*SLC2A9*	rs8055893	16	*CLEC16A*	10.3	3.39	1	MICROS
rs1014290	4	*SLC2A9*	rs1003603	16	*CLEC16A*	10.84	4.27	16	MICROS
rs1014290	4	*SLC2A9*	rs3901386	16	*CLEC16A*	11.05	4.43	16	MICROS
rs1014290	4	*SLC2A9*	rs725613	16	*CLEC16A*	10.71	4.14	13	MICROS
rs1014290	4	*SLC2A9*	rs998592	16	*CLEC16A*	10.89	4.05	15	MICROS
rs733175	4	*SLC2A9*	rs1003603	16	*CLEC16A*	7.13	3.48	11	MICROS
rs733175	4	*SLC2A9*	rs3901386	16	*CLEC16A*	7.1	3.4	11	MICROS
rs733175	4	*SLC2A9*	rs725613	16	*CLEC16A*	7.87	4.25	10	MICROS
rs733175	4	*SLC2A9*	rs2041670	16	*CLEC16A*	7.5	3.57	9	MICROS
rs737267	4	*SLC2A9*	rs8059260	16	*CLEC16A*	15.11	3.83	4	CROATIAN
rs737267	4	*SLC2A9*	rs2302558	16	*CLEC16A*	14.99	3.96	3	CROATIAN
rs737267	4	*SLC2A9*	rs8055893	16	*CLEC16A*	14.32	3.45	2	CROATIAN
rs737267	4	*SLC2A9*	rs9940096	16	*CLEC16A*	15.14	4.2	2	CROATIAN
rs737267	4	*SLC2A9*	rs741176	16	*CLEC16A*	15.56	4.5	2	CROATIAN
rs13131257	4	*SLC2A9*	rs1347591	16	*NUP93*	11.1	4.26	7	MICROS
rs6449213	4	*SLC2A9*	rs1347591	16	*NUP93*	10.51	3.33	3	MICROS
rs4505821	4	*SLC2A9*	rs12149031	16	*NUP93*	7.2	3.46	3	CROATIAN
rs13129697	4	*SLC2A9*	rs10502421	18	*C18orf1*	12.18	3.52	1	MICROS
rs737267	4	*SLC2A9*	rs2042743	18	*C18orf1*	11.99	3.34	14	MICROS
rs6449213	4	*SLC2A9*	rs2042743	18	*C18orf1*	11.01	3.55	6	MICROS
rs1014290	4	*SLC2A9*	rs1540080	18	*C18orf1*	16.61	3.59	10	CROATIAN
rs1014290	4	*SLC2A9*	rs10775489	18	*DYM*	10.35	3.45	3	MICROS
rs1014290	4	*SLC2A9*	rs1838962	18	*DYM*	10.29	3.3	4	MICROS
rs1014290	4	*SLC2A9*	rs1893528	18	*DYM*	10.24	3.4	4	MICROS
rs1014290	4	*SLC2A9*	rs4939846	18	*DYM*	10.72	3.9	3	MICROS
rs1014290	4	*SLC2A9*	rs498929	18	*DYM*	10.39	3.78	4	MICROS
rs1014290	4	*SLC2A9*	rs478682	18	*DYM*	10.14	3.49	5	MICROS
rs733175	4	*SLC2A9*	rs1838962	18	*DYM*	7.73	3.65	2	MICROS

rs733175	4	*SLC2A9*	rs1893528	18	*DYM*	7.39	3.52	2 MICROS
rs733175	4	*SLC2A9*	rs4939846	18	*DYM*	7.47	3.61	2 MICROS
rs733175	4	*SLC2A9*	rs498929	18	*DYM*	7.73	4.04	3 MICROS
rs10805346	4	*SLC2A9*	rs9964222	18	*DYM*	11.51	3.34	3 CROATIAN
rs10805346	4	*SLC2A9*	rs16950548	18	*DYM*	11.54	3.34	3 CROATIAN
rs13129697	4	*SLC2A9*	rs355331	18	*INO80C*	10.75	3.37	11 MICROS
rs13129697	4	*SLC2A9*	rs355338	18	*INO80C*	10.77	3.45	11 MICROS
rs737267	4	*SLC2A9*	rs3786394	18	*INO80C*	15.19	4.04	1 CROATIAN
rs2867394	4	*SLC2A9*	rs1861906	18	*WDR7*	7.07	4.42	5 MICROS
rs13129697	4	*SLC2A9*	rs2278495	18	*WDR7*	10.95	3.38	1 MICROS
rs13129697	4	*SLC2A9*	rs1370215	18	*WDR7*	10.92	3.3	1 MICROS
rs733175	4	*SLC2A9*	rs9946253	18	*WDR7*	11.28	3.68	2 CROATIAN
rs733175	4	*SLC2A9*	rs1437073	18	*WDR7*	11.18	3.5	3 CROATIAN
rs733175	4	*SLC2A9*	rs2083020	18	*WDR7*	11.17	3.53	3 CROATIAN
rs1014290	4	*SLC2A9*	rs9976793	21	*DSCAM*	10.74	3.95	3 MICROS
rs1014290	4	*SLC2A9*	rs909185	21	*DSCAM*	10.39	3.69	4 MICROS
rs733175	4	*SLC2A9*	rs9976793	21	*DSCAM*	7.67	3.84	3 MICROS
rs733175	4	*SLC2A9*	rs909185	21	*DSCAM*	7.65	3.9	3 MICROS
rs10805346	4	*SLC2A9*	rs2837381	21	*DSCAM*	12.12	3.31	73 CROATIAN
rs733175	4	*SLC2A9*	rs2822636	21	*HSPA13*	7.48	3.57	2 MICROS
rs13129697	4	*SLC2A9*	rs2205254	21	*HSPA13*	16.52	3.52	7 CROATIAN
rs737267	4	*SLC2A9*	rs3747208	22	*PARVG*	11.18	3.37	1 MICROS
rs4447863	4	*SLC2A9*	rs139210	22	*PARVG*	7.18	4.15	33 CROATIAN
rs1014290	4	*SLC2A9*	rs713745	22	*SEZ6L*	10.26	3.53	7 MICROS
rs733175	4	*SLC2A9*	rs5761435	22	*SEZ6L*	7.36	3.47	4 MICROS
rs733175	4	*SLC2A9*	rs2073258	22	*SEZ6L*	8.6	4.98	3 MICROS
rs733175	4	*SLC2A9*	rs713745	22	*SEZ6L*	8.85	5.18	4 MICROS
rs733175	4	*SLC2A9*	rs137180	22	*SEZ6L*	10.09	6.58	4 MICROS
rs737267	4	*SLC2A9*	rs1941126	22	*SEZ6L*	14.43	3.74	21 CROATIAN
rs737267	4	*SLC2A9*	rs4822711	22	*SEZ6L*	14.83	3.82	21 CROATIAN

SNP_1 (SNP_2): the first (second) SNP name;

chr_1 (chr_2): the chromosome where SNP_1 (SNP_2) locates

$gene_1$ ($gene_2$): symbol of the gene annotated by SNP_1 (SNP_2);

P_{pair}: $-\log_{10}$ P value of the whole pair test;

$P_{int:}$ $-\log_{10}$ P value of the interaction test;

MGC: count of number of individuals in the minor joint genotype class

Epistatic pairs replicated at the SNP level are highlighted in red

Epistatic pairs involving two shared genes are colored in orange
